# S09-3 Evidence of the impact of Sport Policies on physical activity and sport participation: A Systematic Mixed Studies Review

**DOI:** 10.1093/eurpub/ckac093.047

**Published:** 2022-08-29

**Authors:** Kevin Volf, Liam Kelly, Enrique García Bengoechea, Bláthín Casey, Peter Gelius, Sven Messing, Sarah Forberger, Jeroen Lakerveld, Nicolette R Den Braver, Joanna Zukowska, Catherine Woods, on behalf of the PEN Consortium

**Affiliations:** 1 Department of Physical Education and Sport Sciences, University of Limerick; 2 Physical Activity for Health Research Cluster, Health Research Institute, University of Limerick; 3 Research & Innovation Unit, Sport Ireland, Dublin, Ireland; 4 Department of Sport Science and Sport, Friedrich-Alexander-Universität Erlangen-Nürnberg, 91058 Erlangen, Germany; 5 Leibniz Institute for Prevention Research and Epidemiology – BIPS, 28359 Bremen, Germany; 6 Amsterdam UMC, VU University Amsterdam, Department of Epidemiology and Data Science, Amsterdam Public Health Research Institute, De Boelelaan 1089a, Amsterdam 1081 HV, The Netherlands; 7 Upstream Team, www.upstreamteam.nl, De Boelelaan 1089a, Amsterdam 1081 HV, The Netherlands; 8 Faculty of Civil and Environmental Engineering, Gdansk University of Technology, Narutowicza 11, Gdansk 80-233, Poland

**Keywords:** physical activity, benchmarking, policy, implementation, sport, transport, mass media

## Abstract

**Background:**

Sport is recognised as a potential public health intervention through its influence on physical activity (PA) levels and consequent health benefits. International policy actors such as the Council of Europe, the World Health Organisation and the International Society for Physical Activity and Health have recommended that sport for all is promoted both for public health and as a basic right.

Purpose: This review aims to provide evidence to support the development of policies aimed at maximising the opportunity to participate in PA and sporting activity.

**Methods:**

We systematically searched six electronic databases for quantitative, qualitative, and review studies investigating how public sport policy affects PA and sport participation outcomes”. The scientific literature was screened according to predetermined eligibility criteria. Following study selection and data extraction, the quality was assessed using modified versions of existing quality assessment tools. Results were synthesised narratively.

**Results:**

Database searches identified 3705 unique articles. A total of 93 full-text articles were assessed, with 31 meeting our inclusion criteria. Fourteen unique policy actions were identified and were categorised into the policy areas Build Sport Facilities, Reduce Financial Barriers, Build Capacity and Establish Partnerships with Sport Sector, and Promote Public Interest in Sports.

**Conclusions:**

Policy actions to promote physical activity and sport participation have demonstrated qualified success but there is limited evidence of success in reaching hard to reach groups. Therefore, policymakers utilising sport to increase physical activity should treat it as a complementary intervention alongside other policy actions based on a systems perspective.

